# New Functional Criterion for Evaluation of Homologous MDR Pumps

**DOI:** 10.3389/fmicb.2020.592283

**Published:** 2020-11-11

**Authors:** Pavel A. Nazarov, Alexandra I. Sorochkina, Marina V. Karakozova

**Affiliations:** ^1^Department of Bioenergetics, Belozersky Institute of Physico-Chemical Biology, Lomonosov Moscow State University, Moscow, Russia; ^2^Laboratory of Molecular Genetics, Moscow Institute of Physics and Technology, Dolgoprudny, Russia; ^3^Center of Life Sciences, Skolkovo Institute of Science and Technology, Moscow, Russia

**Keywords:** paralog, antibacterial drug, AcrAB-TolC, SkQ1, multidrug pump

## Introduction

In recent years, bacterial resistance has become increasingly important for health care. With a simultaneous decrease in the number of newly registered antibacterial drugs, resistance to them is growing at an ever-higher rate, which makes the development of new drugs an expensive and ineffective undertaking. Therefore, creation of effective new antibacterial drugs is the most important task of modern Drug Development. Recently, several antibacterial drugs have been discovered (Khailova et al., 2015; Ling et al., 2015; Dibrov et al., 2017; Nazarov et al., 2017; Imai et al., 2019; Luther et al., 2019); however, for all of them, there are restrictions on the spectrum of action on bacteria. Some of them are active only toward gram-negative bacteria (Imai et al., 2019; Luther et al., 2019), while others toward gram-positive (Ling et al., 2015). However, for one of them (Khailova et al., 2015; Nazarov et al., 2017), the spectrum of action opened up as the antibiotic was being studied, from acting only toward gram-positive bacteria (Khailova et al., 2015), to acting on any bacteria, with the exception of those which had a certain AcrAB-TolC multidrug resistance pump (Nazarov et al., 2017, 2019).

The tripartite efflux system AcrAB-TolC is the main drug efflux transporter complex in *Escherichia coli* (Tam et al., 2020), which extrudes multiple antibiotics (erythromycin, oxacillin, ciprofloxacin, etc.), dyes (rhodamine 6G, ethidium, acridine, etc.), bile salts, detergents (SDS, taurocholate, berberine, tetraphenylphosphonium, etc.) and small organic molecules (hexane, indole, cyclohexane, etc.) (Pos, 2009). AcrAB–TolC is comprised of the outer membrane protein TolC, the periplasmic adaptor protein AcrA, and the inner membrane transporter AcrB from the resistance-nodulation-cell division (RND) superfamily (Shi et al., 2019).

**Figure 1:**
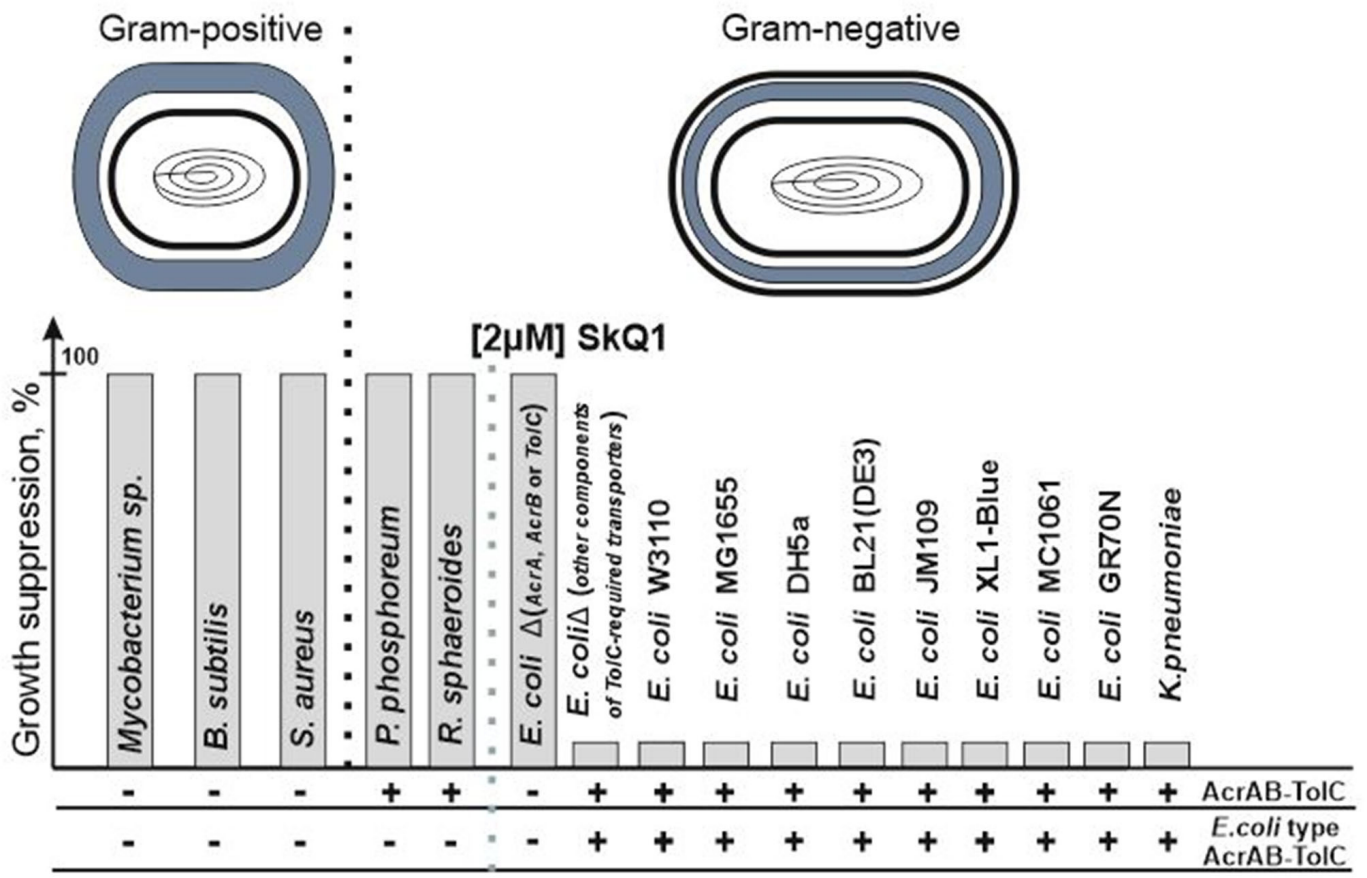
SkQ1 resistance does not depend on the structure of the cell wall. The presence of all genes encoding AcrAB-TolC pump's proteins in the genome (AcrAB-TolC) and the presence of proteins similar (the identity is higher than the identity between the sequences of AcrD and AcrB proteins) to those from *E. coli* (*E. coli* type AcrAB-TolC) among them are marked as (+). Since AcrAB-TolC deletion mutants do not contain all proteins of the AcrAB-TolC pump and therefore the pump cannot be assembled in their cells, these bacteria are designated as (–). Deletions of genes encoding other TolC-containing pumps' proteins (for example, the AcrD gene from the AcrAD-TolC pump or the MdtE/MdtF genes from the MdtEF-TolC pump) did not lead to growth suppression.

Mitochondria-targeted antioxidant SkQ1 (decyl triphenylphosphonium-conjugated plastoquinone) is a member of a new class of antibiotics that directly affect bacterial bioenergetics. The use of synthetic non-targeted compounds has its own advantages and seems to be quite promising (Hards and Cook, 2018; Nazarov, 2018). SkQ1 is one of the most researched mitochondria-targeted antioxidants, and although the protective effect of SkQ1 and SkQR1 in acute bacterial infection has been well studied in an *in vitro* inflammation model and in an *in vivo* rat model of acute pyelonephritis in the presence of bacterial lysate (Plotnikov et al., 2013), it was believed that SkQ1 lacks antibiotic properties (Anisimov et al., 2011). However, although no antibacterial effect was observed against the classical model gram-negative bacterium *Escherichia coli*, an antibacterial effect was observed against another classical model gram-positive bacterium *Bacillus subtilis* (Khailova et al., 2015). In further studies (Nazarov et al., 2017), it was found that SkQ1 is effective as an antibacterial agent toward all the studied gram-positive bacteria, regardless of the structure of the bacterial cell envelope. Moreover, upon removal of any of the proteins of the AcrAB-TolC pump in *E. coli*, the bacterium completely lost its resistance against SkQ1 which became comparable to that of gram-positive bacteria (Figure 1). It should be noted that, according to modern concepts, the AcrAB-TolC pump contains not only the AcrA, AcrB, and TolC proteins, but also the small accessory protein AcrZ (Hobbs et al., 2012; Du et al., 2014). However, in the case of resistance to SkQ1, the deletion of the *acrZ* gene did not affect the resistance of *E. coli* (Nazarov et al., 2019). This suggested that the resistance depends on the presence of the AcrAB-TolC pump, but this observation turned to be preliminary. At this time, however, we can confirm that SkQ1 is expelled from cells by only one multidrug resistance pump under normal conditions. This fact makes SkQ1 an interesting and effective tool for studying pump performance, especially that of TolC-containing pumps.

## SkQ1-Based Analysis of Proteins Comprising TolC-Containing Pumps

In our studies (Nazarov et al., 2017, 2019), we analyzed all pumps containing TolC in relation to SkQ1 under conditions close to physiological in the cell. Although the important contribution of protein topogenesis and quality control of protein complexes to mitochondrial function is known (Luzikov, 2009), the topogenesis of bacterial membrane proteins is only beginning to be intensively studied (Mercier et al., 2020). However, the quality control of the assembled complexes is often not fully understood, therefore the effect of the absence of a pump component and its contribution to the formation of the bacterial membrane infrastructure cannot be precisely estimated. For example, deletion of TolC is known to cause changes in the expression of a number of membrane proteins, such as OmpF (Rosner and Martin, 2009) and can lead to metabolic shut-down (Dhamdhere and Zgurskaya, 2010). However, for most proteins of TolC-containing pumps, the effect on the phenotype has not been adequately studied.

Analysis of deletion mutants showed that loss of resistance occurs in the case of deletion of any of the proteins of the AcrAB-TolC pump (except AcrZ), thereby confirming the dependence of the resistance on the presence of the AcrAB-TolC pump in the bacterial membrane. Another important finding was the fact that under physiological conditions, none of the remaining pumps replaced the AcrAB-TolC function in its absence. This strikingly distinguishes bacterial pumps from eukaryotic ones, where pleiotropy is observed (Knorre et al., 2014), which may be one of the reasons for the greater resistance of both yeast and other eukaryotic cells to SkQ1.

Another important conclusion is the confirmation of the necessity of the AcrA protein for the functioning of the AcrAB-TolC pump. Although TolC protein interacts with both AcrA protein and AcrB protein (Symmons et al., 2009) and TolC can bind AcrB directly, in accordance with the current paradigm, the formation of the tripartite complex is initiated by TolC-AcrB binding and only stabilized by AcrA binding (Tikhonova et al., 2011), but without AcrA, the complex does not work properly. This observation is the first confirmation of the necessity of the AcrA protein in the formation of an active AcrAB-TolC pump. Moreover, this apparently excludes a significant role of the amino acid residues of the AcrA and TolC proteins in the formation of the active center of the AcrAB-TolC pump, since the AcrAD-TolC pump containing AcrA, TolC, and AcrD protein homologous to AcrB protein (66% sequence identity) is unable to pump out SkQ1 from bacterial cells (Nazarov et al., 2019).

## All *Escherichia coli* Strains Demonstrated Identical Resistance to SkQ1

*Escherichia coli* strains are the most studied bacterial species with over a thousand complete or partial genome sequences, making them excellent subjects for analysis. Many of the *E. coli* strains have a long history of laboratory evolution and have undergone random or targeted mutagenesis. Strain B was described by d'Herelle at the Pasteur Institute in Paris in 1918 and strain K-12 was first isolated at Stanford University in 1922 (Karakozova and Nazarov, 2018). The genes coding for multidrug resistance pumps, such as AcrAB-TolC, are not essential, so multiple point mutations, partial or complete deletions are expected to occur in this class of genes. However, when analyzing the sequences of different laboratory strains, attention is drawn to the fact that the sequences of all three proteins AcrA, AcrB and TolC are identical for all strains and do not contain even a single substitution, despite the fact that the strains were isolated on different continents, countries, cities and research institutes (Karakozova and Nazarov, 2018). This suggests that we can assume identical resistance for all studied strains, which was confirmed in laboratory studies (Nazarov et al., 2019). Thus, despite the fact that the genes of the AcrAB-TolC pump are not essential, natural selection is aimed at maintaining the conservation of the amino acid sequences of these proteins, apparently due to their important function of clearing the bacterial cell from toxic components and the involvement of the AcrAB-TolC pump in the continuous “cleaning” of bacterial cells (Karakozova and Nazarov, 2018). Thus, significant substitutions in the sequences may lead to the loss of important functions in cell physiology of *E. coli* bacteria. This observation was confirmed in a recent work (Bhattacharyya et al., 2020), which demonstrated the key role of individual amino acid residues of AcrA and TolC in “necrosignal” recognition that activates swarm-specific resistance in an *E. coli* population. Thus, it can be assumed that the AcrAB-TolC pump may not only remove certain molecules from the cell, but also is a keystone element in regulatory processes important for the physiology of bacteria.

On the other hand, the nature of bacterial resistance to SkQ1 makes it possible to compare the level of resistance in independent experiments under different experimental conditions, normalizing the resistance to that of the *E. coli* strains.

## AcrAB-TolC Pump Does Not Ensure SkQ1 Resistance

We might assume that the presence of the AcrAB-TolC pump means that cells will be resistant to SkQ1, but this is not the case. Gram-negative bacteria *Rhodobacter sphaeroides* and *Photobacterium phosphoreum* that demonstrated sensitivity to SkQ1 also contained an AcrAB-TolC pump. This seeming paradox turned out to be easy to explain. AcrB protein sequences from *Rhodobacter sphaeroides* and *Photobacterium phosphoreum* demonstrate only 33 and 65% identity, respectively, to the AcrB protein sequence from *E. coli* (Nazarov et al., 2019). As mentioned above, sequence identity between AcrB and AcrD proteins is 66%, but the AcrAD-TolC pump cannot substitute for the AcrAB-TolC pump ability to remove SkQ1 from cells. It should be emphasized that when analyzing the entire tripartite complex, the overall sequence similarity between AcrAB-TolC pumps from different bacteria will decrease due to the difference in the sequences of other proteins (AcrA and TolC), while when comparing AcrAB-TolC and AcrAD-TolC from *E. coli*, the overall sequence similarity will increase. This allows us to assume that with <66% sequence identity of the AcrB protein, resistance to SkQ1 cannot be expected.

## Discussion

It is known that a conclusion that two or more genes or proteins are homologous is a conjecture, not an experimental fact (Koonin and Galperin, 2003). We can talk about homology, similarity, sequence identity, but the most important thing for comparing two protein pumps is whether we can say that they are functionally identical. A 33% identity may look quite significant, and if it met the criteria for homologous sequences (Koonin and Galperin, 2003), and the similarity extended over a long stretch of sequence, we would say that these are homologous protein sequences. However, we would not be able to say that these were functionally identical proteins because we missed one very important criterion – that of functional identity. It is clear that pumps can function on a variety of substrates, and sequence changes can and appear to lead to changes in substrate specificity. While this is a relatively rare documented property, pumps appear to have a set of unique substrates, by which we mean a molecule or a group of molecules with similar structural patterns that are recognized by only one pump in a cell. If a unique pump substrate is available, a functionally identical pump will remove it regardless of its sequence identity. Lack of substrate specificity for unique substrates will indicate that the pump is no longer the same. Since SkQ1 is a unique substrate for AcrAB-TolC, the main MDR pump of *E. coli*, the loss of the ability to recognize and remove SkQ1 by bacteria is an evidence of the lack of a functional AcrAB-TolC pump. The ability to recognize and remove a unique substrate, however, does not guarantee the availability of a functional pump, and requires sequence comparison and application of criteria for sequence analysis (Koonin and Galperin, 2003).

However, what if SkQ1 was a unique substrate only for the *E. coli* AcrAB-TolC pump, and this was not applicable to other bacteria? Then we would observe resistance in the *E. coli* bacteria and sensitivity in all other gram-negative bacteria, as in the case of *R. sphaeroides* and *P. phosphoreum*. However, this is not supported by evidence, and the bacteria *Klebsiella pneumoniae* having an identity of 91.5% also demonstrates resistance comparable to *E. coli* (Figure 1) (Nazarov et al., 2019), which confirms the existence of a previously missing criterion for functionally homologous proteins.

Thus, proteins AcrB and AcrD from *E. coli* are genetic and functional paralogs, and proteins AcrB from *E. coli* and *K. pneumoniae* are genetic and functional orthologs. In turn, the AcrB proteins from *E. coli, R. sphaeroides*, and *P. phosphoreum* may be genetically orthologous, but functionally they are paralogs. Therefore, the presence of a similar pump cannot be a necessary and sufficient condition for establishing the presence of resistance for bacteria and the functional test is required.

## Author Contributions

PN: funding acquisition. PN, AS, and MK: writing. All authors have read and agreed to the published version of the manuscript.

## Conflict of Interest

The authors declare that the research was conducted in the absence of any commercial or financial relationships that could be construed as a potential conflict of interest.
